# The FAT expandability (FATe) Project: Biomarkers to determine the limit of expansion and the complications of obesity

**DOI:** 10.1186/s12933-015-0203-6

**Published:** 2015-04-22

**Authors:** Elena Torres-Perez, Monica Valero, Beatriz Garcia-Rodriguez, Yolanda Gonzalez-Irazabal, Pilar Calmarza, Luisa Calvo-Ruata, Carmen Ortega, Maria Pilar Garcia-Sobreviela, Alejandro Sanz-Paris, Jose Maria Artigas, Javier Lagos, Jose M Arbones-Mainar

**Affiliations:** Adipocyte and Fat Biology Laboratory (AdipoFat), Unidad de Investigación Traslacional, Hospital Universitario Miguel Servet, Instituto Aragonés de Ciencias de la Salud (IACS), Zaragoza, Spain; Unidad de Cirugía, Hospital Royo Villanova, Zaragoza, Spain; Servicio de Bioquímica Clínica, Hospital Universitario Miguel Servet, Zaragoza, Spain; Servicio de Endocrinología y Nutrición, Hospital Universitario Miguel Servet, Zaragoza, Spain; Servicio de Radiodiagnóstico, Hospital Universitario Miguel Servet, Zaragoza, Spain; Instituto de Investigación Sanitaria Aragón (IIS Aragón), Zaragoza, Spain; Centro de Investigación Biomédica en Red Fisiopatología Obesidad y Nutrición (CIBERObn), Instituto Salud Carlos III, Madrid, Spain

**Keywords:** Adipose tissue, Biomarkers, Type 2 diabetes, CT-scan, Metabolomics

## Abstract

**Background:**

Obesity is an excessive accumulation of fat frequently, but not always, associated with health problems, mainly type 2 diabetes and cardiovascular disease. During a positive energy balance, as caused by excessive intake or sedentary lifestyle, subcutaneous adipose tissue expands and accumulates lipids as triglycerides. However, the amount of adipose tissue *per se* is unlikely to be the factor linking obesity and metabolic complications. The expandability hypothesis states that, if this positive energy balance is prolonged, a point is eventually reached where subcutaneous adipose tissue can not further expand and energy surplus no longer can be safely stored. Once the limit on storage capacity has been exceeded, the dietary lipids start spilling and accumulate ectopically in other organs (omentum, liver, muscle, pancreas) forming lipid byproducts toxic to cells.

**Methods/Design:**

FATe is a multidisciplinary clinical project aimed to fill gaps that still exist in the expandability hypothesis. Imaging techniques (CT-scan), metabolomics, and transcriptomics will be used to identify the factors that set the limit expansion of subcutaneous adipose tissue in a cohort of caucasian individuals with varying degrees of adiposity. Subsequently, a set of biomarkers that inform the individual limits of expandability will be developed using computational and mathematical modeling. A different validation cohort will be used to minimize the risk of false positive rates and increase biomarkers' predictive performance.

**Discussion:**

The work proposed here will render a clinically useful screening method to predict which obese individuals will develop metabolic derangements, specially diabetes and cardiovascular disease. This study will also provide mechanistic evidence that promoting subcutaneous fat expansion might be a suitable therapy to reduce metabolic complications associated with positive energy balance characteristic of Westernized societies.

## Background

Obesity is a chronic disease of multifactorial origin and physiologically defined as an accumulation of fat that causes health problems. At the population level, this excessive adiposity has been epidemiologically associated with a wide array of metabolic complications such as cardiovascular disease, type 2 diabetes, and some types of cancer [1-4]. At the individual level, however, disparities do exist; some thin people are insulin resistant, while some very obese people remain metabolically healthy on the basis of a beneficial adipose tissue growth [5-9]. The amount of adipose tissue *per se* is hence unlikely to be the factor linking obesity and metabolic complications.

Adipose tissue is located under the skin (subcutaneous) or within the abdominal wall (visceral) and endowed with a high plasticity, being able to expand and contract in response to changes in energy balance. Currently it is believed that the accumulation of subcutaneous fat is metabolically harmless. The expansion of subcutaneous fat is determined by the formation of new adipocytes and the ability to expand from those already formed [10]. The new adipocytes appear from their precursors, known as preadipocytes, vascular stroma, as well as adipose or mesenchymal stem cells [11]. The total number of stem cells available to differentiate into new adipocytes determines the intrinsic limit in the adipose tissue expansion, given the inability to form new adipocytes once this "pool" of precursors is exhausted [12]. This growth process is also based in the ability to remodel the extracellular matrix that surrounds the adipocytes and the formation of new vessels (angiogenesis) irrigating the new cells [13]. This expansive process is hence regulated by the coordinated expression of genes, proteins, and metabolites from different cell types. The recently developed theory of the expandability of adipose tissue states that this expansion is not unlimited. At some point, adipose tissue reaches its maximum storage capacity and lipid excess is redirected to other tissues causing lipotoxicity [14,15]. This ectopic accumulation in different organs and, among them, the peritoneum, is associated with a chronic inflammatory condition of low intensity. Macrophages, lymphocytes, eosinophils, natural killer (NK) and mast cells are known to dwell in the adipose tissue, yet their precise role needs to be elucidated [16-18].

The work proposed here will help fill gaps that still exist in this theory and define the factors that limit expansion. Using high-throughput technologies, this study aims to discover novel mechanisms regulating the expansion of subcutaneous adipose and how they are involved in the development and progression of metabolic complications occurring in obesity. Subsequently, a set of biomarkers that inform the individual limits of expandability will be developed using computational and mathematical modeling.

## Methods

### Hypothesis

During a positive energy balance, as caused by excessive intake or sedentary lifestyle, subcutaneous adipose tissue expands and accumulates lipids as triglycerides. We hypothesize that, if this situation is prolonged, a point is eventually reached in the subcutaneous adipose tissue where it can not further expand and energy surplus no longer can be safely stored. Once the limit on storage capacity has been exceeded, the dietary lipids start spilling and ectopically accumulating in other organs (omentum, liver, muscle, pancreas), giving rise to lipid byproducts that are toxic to cells. Thus, adipose tissue has a defined growth boundary for a given individual, and this limit has a large inter-individual variability.

### Objectives

FATe is a clinical project with the following specific aims: 1) Use of imaging techniques (CT-scan), metabolomics, and transcriptomics to identify the factors that set the limit expansion of subcutaneous adipose tissue in individuals with varying degrees of adiposity. 2) use those factors to build biomarkers able predict the onset and severity of the metabolic complications of obesity. 3) gather a collection of biological samples (serum, plasma, adipose biopsies, adipose RNA, and DNA) for ancillary studies to identify new genetic and environmental determinants of the association of obesity and metabolic abnormalities.

### Determination of fat expansion

**Figure 1:**
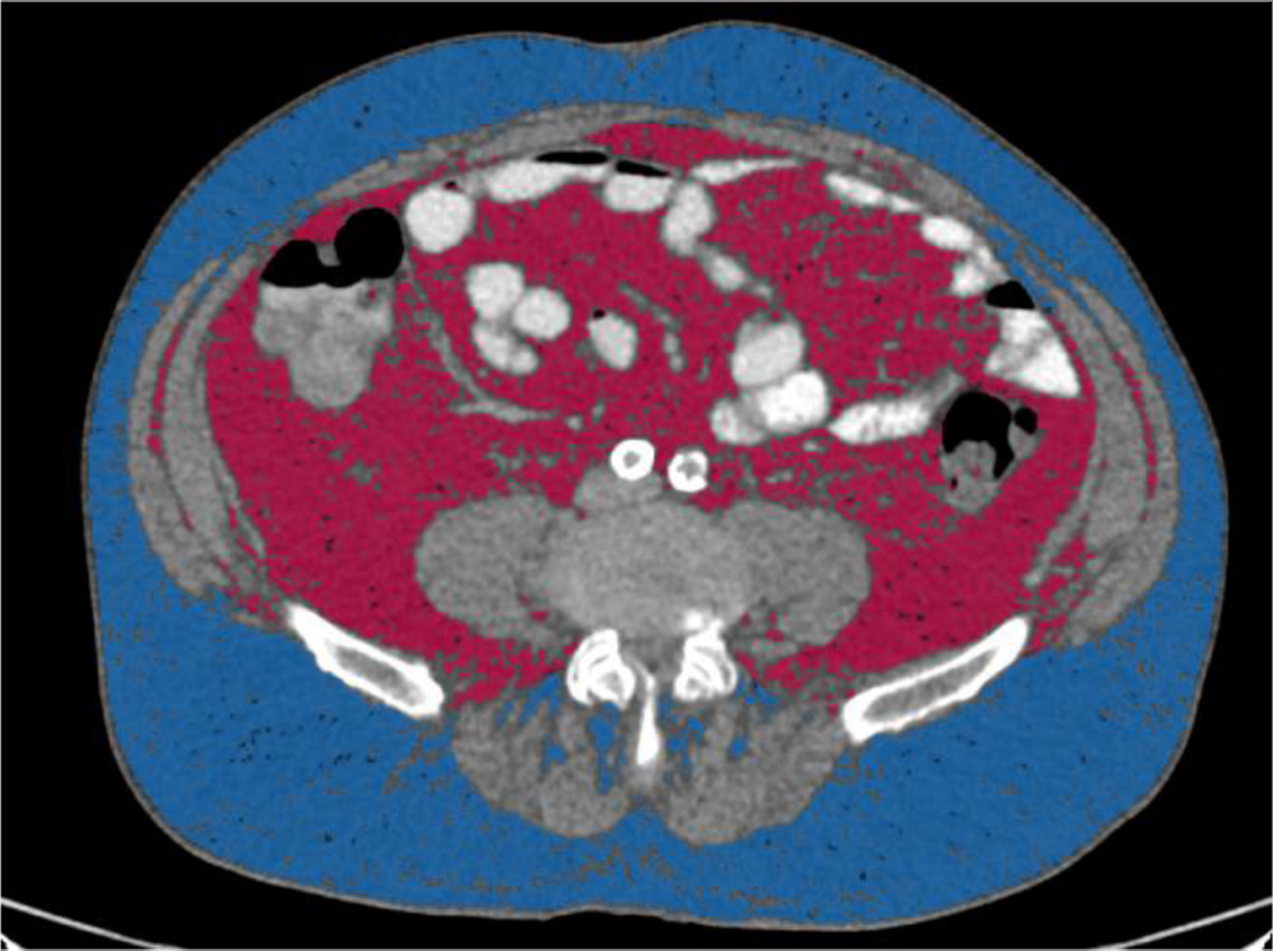
Abdominal adipose tissue segmentation. Representative CT scan obtained at the umbilicus. Subcutaneous and visceral fat are colored in blue and red, respectively.

The visceral and subcutaneous fat will be measured by computer tomography (CT) with a 8mm single slice at the umbilical level. All CT examinations will be acquired with the subject positioned supine in a 64 detector CT scanner (Aquilion 64 Toshiba Tokyo, Japan) and tube voltage set to 120 kVp with automatic tube current modulation and rotation time of 0,5 s. Acquired images will be then transferred to a workstation and analyzed with the Vitrea CT Fat Measurement software (Vital Imaging Inc. The Netherlands). Selected fat densities will range between −150 and −70 Hounsfield Units (HU) and the Total Fat Area (TFA), Subcutáneous Fat Area (SFA) and Visceral Fat Area (VFA) will be measured in cm^2^. SFA and VFA are respectively defined as pixels (area) located outside or inside the outer surface of the abdominal muscle wall (Figure 1). Subsequently, the ratio between subcutaneous fat area divided by the area of visceral fat will be calculated. Operationally, lower VFA/TFA ratios due to increased visceral lipid deposition will be surrogates of limited ability of adipose subcutaneous to further expand.

### Subject participation

#### Ethics, consent and permissions

All patients will provide written consent according to the Spanish 41/2002 law and the the study has been approved by the by the Regional Institutional Review Board of Aragón. Spain. (CEIC-A).

#### Prospective study

**Figure 2:**
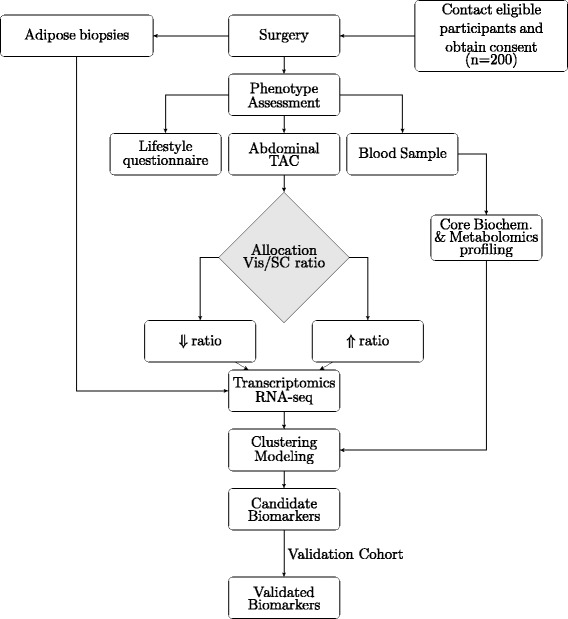
Flowchart for the FATe study.

The study will take place in the Hospital Universitario Miguel Servet (HUMS) and the Hospital Royo-Villanova (HRV), both in Zaragoza (Spain). As illustrated in the flowchart (Figure 2), patients scheduled for laparoscopic surgery will be offered the opportunity to participate in the study by donating blood, biopsies of subcutaneous adipose tissue (also visceral if the type of surgery allows) and undergo a CT imaging test for the quantitative determination of subcutaneous and visceral abdominal fat depots. The inclusion and exclusion criteria are shown in Table 1. We have estimated to enroll 200 patients (150 bariatric patients) over 18 months. Biopsies (~3 cm^3^) of adipose tissue from both the subcutaneous fat depot and the great omentum will be obtained with a bipolar/ultrasonic device (Thunderbeat. Olympus, Spain) and extracted via a 12 mm trocar (Applied Medical Europe, Spain) inserted in the left hypochondrium during laparoscopic surgery. Later on, subjects will continue to receive their usual care by their primary physician and endocrinologist with close oversight by our research team during the first year.

**Table 1:** **Selection criteria**

**Inclusion criteria**	**Exclusion criteria**
● Scheduled (non-emergency) and laparoscopic bariatric surgery, cholecystectomy surgery, surgical treatment of esophageal hiatal hernia, and abdominal hernia repair.	● Alcohol abuse
	● Autoimmune diseases
	● Present history of malignancies
	● Chronic inflammatory diseases
	● Chronic infectious diseases (HIV, HBV, HCV)
● Willingness to participate in the study and comply with the study by signing a written informed consent	
● Available for follow-up visits over 1 year for the prospective cohort and 4 years for the validation cohort.	

#### Validation study

Cohort of 130 patients with the same selection criteria who underwent the same procedures than the prospective cohort. Blood and fat biopsies have already been obtained and the individuals extensively phenotyped. As validation cohort this subjects will be annually evaluated in the Endocrinology Service of the HUMS to study its evolution during this period, with special emphasis in diabetes onset and cardiovascular disease.

#### Characterization of cell heterogeneity by flow cytometry

To characterize different cell populations within the adipose tissue, fresh fragments of both visceral and subcutaneous adipose tissue biopsies will be digested with collagenase to obtain a cell suspension. Cell size and lipid content *per* adipocyte will be assessed as previously described [19]. At least 100,000 cells in PBS with 0.5% BSA and 2 mmol/L EDTA will be incubated with fluorescent-labeled monoclonal antibodies directed against angiogenic, endothelial, and immune cell markers as described [20,21]. After washing steps, the labeled cells will be analyzed in a GALLIOS flow cytometer with the KALUZA software (Beckman Coulter). Mesenchimal stem cells will be considered those expressing the CD105, CD73 and CD90 antigens and not expressing hematopoietic cell markers CD45 and CD34.

### Variables and determinations

#### Anthropometry and questionnaires

The variables sex, age, height, and blood pressure will be collected at the time of the preoperative visit. Likewise, a questionnaire will be used to asses socio-demographic data, smoking history, consumption of alcohol or other toxic habits, previous medical diagnosis, presence of comorbidities, family history of disease, and medication use. Clinical records of each patient will be also evaluated to determine the criteria for inclusion/exclusion.

#### Plasma biochemistry

Standard operating procedures will be deployed to assure identical collection and processing for all fasting blood samples. Extensive bloodwork will be performed at the Clinical Biochemistry Service (HUMS) using state of the art analyzers (Table 2). All analyses are in compliance with the requirements for quality and competence (ISO 15189:2012) for medical laboratories.

**Table 2:** **Core laboratory parameters**

**Origen**	**Parameter**
Serum	Total cholesterol, Triglycerides, LDL-cholesterol, HDL-cholesterol, Free fatty acids
	Glucose, Insulin, β-hidroxibutirate, hs-CRP, Leptin
	Liver Transaminases (AST, ALT, GGT)
	Apolipoprotein AI, Apolipoprotein B, Lipoprotein (a)
	25-Hydroxyvitamin D, Beta-CrossLaps (CTX), Osteocalcin
	Selenium
Total blood	Hematimetry (Complete blood count)
	Glycated Hemoglobin (HbA1c)

#### Metabolomics

Plasma aminoacid concentrations will be measured by ion-exchange chromatography (IEC) with ninhydrin detection. Briefly, plasma samples (1 ml) will be deproteinized with 0.1 ml of 35% sulfosalicilic acid (Panreac, Spain) followed by centrifugation. Supernatants will be then filtered trough 0.22 μm (Ultrafree Centrifugal filters. Millipore, USA) and mixed with the same volume of 0.2 M lithium citrate buffer, pH 2.2. (Biochrom. Germany). 20 μl of this mixture will be injected into a Biochrom +30 system (Biochrom), a high pressure PEEK column packed with Ultropac 8 cation exchange resin. Aminoacids will be eluted by varying temperature, ionic strength and pH of the lithium citrate buffer according to the instrument manufacturer instructions. Detection will be carried out by the reaction of ninhydrin with amino acids to form colored compounds. The color produced will be photometrically detected at 440 nm (primary amines) and 570 nm (secondary amines). BioSys control software and EZChrom Elite Data handling Software (Biochrom) will be used for data acquisition and analysis. Amino acid calibration will be prepared at know concentrations for each analyte with commercially available standards (Sigma) and run every time that fresh ninhydrin is prepared.

Acylcarnitine profiling will be carried out by liquid chromatography tandem mass spectrometry (LC-MS/MS) system. Briefly, dried blood spots will be eluted from filter paper with extraction solvent containing methanol and known concentrations of stable isotopically enriched acylcarnitines (NeoBase Non-derivatized Assay Solutions. Perkin Elmer, Finland). The eluate (10 μL) will be loaded into a Waters 2795 HPLC sample preparation system connected to a Micromass Quattro Micro triple-quadrupole mass spectrometer (Waters Corp., USA). The HPLC pump provides a flow of 0.150 mL/min and injection-to-injection time is 1.7 min. The instrument will be operated in the positive ion mode with the following parameters: cone voltage and collision energy will range from 16 to 20 V and from 18 to 28 eV, respectively depending on the analyte, using argon as collision gas. The ion source and desolvation temperatures will be 120 and 350°C, respectively. Data will be acquired using MassLynx 4.0 software (Waters Corp.). During this acquisition, a collisionally induced product of each analyte is measured for a set time period and quantified based on their mass to carge (m/z) ratio. The response of each analyte relative to their corresponding stable-isotope labelled internal standard will be used to quantify analyte concentration.

Plasma and erythrocyte membrane fatty acids will be measured by gas chromatography (GC) analysis. In summary, plasma will be obtained by centrifugation of heparinized blood samples. Cell pellets, mainly erythrocytes, will be washed with saline solution and subsequent fatty acid extraction from both plasma and erythrocytes will be carried out according to the modified Folch [22] method using chloroform, methanol and 0.015M KCl (2:1:0.4, volume to volume (v/v)) (Merk. Spain). Fatty acids in the chloroform phase will be then recovered after evaporation under nitrogen stream followed by methylation with 5% H_2_SO_4_ in methanol at 80°C for 90 min. Fatty acid methyl esters will be extracted into 300 μl hexane and then injected into an Agilent 6890 N equipped with a capillary column HP-INNOWax (ref#19091N-102) and flame ionization detector (both from Agilent Technologies, Spain). Initial temperature for the gas chromatography will be set at 60°C for 2 min, increased to 190°C (30°C/min), held at 200°C for 13 min, and finally increased to 240°C for 25 min. Hewlett-Packard ChemStation System software (Hewlett-Packard, Spain) will be used for identification of individual fatty acids by comparison with known standards (Sigma) and expressed as a percentage of total fatty acids quantified from peak areas.

#### Transcriptomics

RNA sequencing (RNA-seq) analyses will be carried out in subcutaneous biopsies with different ratios of expandability calculated by CT-scan. Samples from subjects with greater ratios (subcutaneous fat still able to expand) will be thus compared to lower ratios (limit of expansion already reached). Total RNA from selected biopsies will be extracted and DNAse-treated. Library preparation will be performed as recommended by the manufacturer. Samples will be sequenced to a coverage of, at least, 40 million single-end reads of 50 bp long. The genes of interest whose expression appears increased or decreased will be confirmed by quantitative PCR (qPCR) using the appropriate primers and SYBR Select Master Mix (Applied Biosystems, USA) in a StepOnePlus Real-Time PCR System (Applied Biosystems).

### Sample biobanking

The Biobank of the Aragon Health System (BSSA) will store all biological samples and their associated data following standardized protocols to ensure their viability and reliability. Samples will be fully accessible to the scientific community, and a laboratory informatics management applications (Bio-e-bank. Vitrosoft, Spain) has been implemented to maintain sample traceability.

### Data collection and analysis

Given i) the project lacks a single main study variable and ii) variance estimates from omics-generated variables are unknown, the forecasted sample size is determined for our team's ability to recruit. Retrospective analysis will be carried out to detect the power of the differences observed in the data.

All the data collected will be incorporated into a database meeting the Spanish Organic Law 15/1999 of Protection of Personal Data (LOPD). Pairwise comparisons between continuous variables will be conducted using either Student's *t* test or nonparametric Mann–Whitney *U* test as appropriate. Chi square tests will be performed for categorical variables. Pearson correlation coefficient or Spearman Rho, according to the homogeneity of the distribution of data, will be calculated to determine correlations. For the RNA-seq analysis, the pre-processing, quality testing, mapping, and differential gene expression will be performed using the appropriate packages of Bioconductor (http://www.bioconductor.org/).

Biomarkers will be a combination of variables, measured in adipose tissue and plasma, generated by the core biochemical analysis, multiplexed cytometry, as well as transcriptomic and metabolomic analysis. Those analytes will be included in a multivariate analysis; cluster analysis, support vector machines and dimensionality reduction will segregate groups that appear differentially expressed depending on the ratios of expandability calculated by CT-scan. Subsequently, those analytes will be modeled individually and in groups using multivariate logistic regression where the independent variable will be whether or not the limit of adipose tissue expansion has been reached. The predictive value of those biomarkers will be calculated using ROC curves in the validation study. Different predictive models adjusted for confounding variables (BMI, age) will be described using appropriate statistical software package R (http://cran.r-project.org/).

## Discussion

We hypothesize that the accumulation of lipids in the adipose tissue, as long as its expansive capacity is preserved, represents an adaptive and safe way to store energy surplus. We accordingly define dysfunctional adipose tissue as not able to expand and adequately store the surplus of lipids provided by a positive energy balance. This dysfunctional adipose tissue ultimately leads to conditions such as metabolic syndrome, diabetes, dyslipidemia, and cardiovascular disease. The FAT expandability (FATe) Project is an observational clinical study aimed to elucidate the mechanisms involved in the expansion of the subcutaneous adipose tissue and find biomarkers to determine the limit of expansion and able to predict complications of obesity.

Mounting evidence supports that the adverse consequences of obesity are strongly associated with the location of fat accumulation, and are less dependent on the total amount of body fat. Visceral obesity correlates with increased risk of type 2 diabetes and cardiovascular diseases, while an increase of subcutaneous fat is associated with favorable adipokine production and improved plasma lipid profiles [23-25]. Indeed, the accumulation of subcutaneous adipose tissue in the gluteofemoral area might be protective against obesity-associated metabolic complications [26]. Consequently, it is of paramount importance to segment this adipose tissue in more defined compartments. The body mass index (BMI, weight in kilograms divided by height in meters squared) is the most widely used method to estimate the amount of body fat. However, since BMI can not detect regional variations in fat deposition, other measures able to capture abdominal obesity, such as waist circumference (WC) or waist/hip ratio (WHR, waist circumference divided by the hip circumference) are also employed. Yet, the use of those indices can not discern the contribution of subcutaneous adipose tissue or visceral, in abdominal girth. The only way to differentiate these two deposits is by imaging techniques. Currently, the only validated techniques for measuring visceral fat are the X-ray computed tomography (CT) or magnetic resonance imaging (MRI) [27]. The longer time needed to perform MRI, compared to CT, led us to selected the latter for this study, given the requirement to combine the use of the scanner for both clinical and research purposes. Our study protocol is designed to minimize the radiation exposure associated with CT without compromising accuracy in estimating abdominal fat depots. Several studies have already implemented similar methodology proving that a single slice is enough to segment abdominal fat by CT [28,29]. Some controversy still remains regarding the point of reference. However, recent work revealed that the measurement of the fat based on an umbilical slice was well correlated and well concordant with that based on a slice with a L3–L4 reference [28].

Biomarker, portmanteau of “biological marker”, refers to any measurable indicator of metabolic processes, ranging from heart rate or basic plasma chemistries (e.g. glucose) to more complex constructs produced by high-throughput techniques [30]. To the best of our knowledge, no specific biomarkers for detection of a maxed-out subcutaneous adipose tissue have been tested for prediction in humans. We will define the calculated ratio between the areas of visceral and subcutaneous adipose tissue as the surrogate variable for fat expandability. The subsequent prospective study groups will be created according to this variable. Then, we will begin the quest for biomarker discovery using a combined transcriptomic and metabolomic approach. This holistic conceptualization has been already proven valuable in deciphering adipose tissue signatures related to weight changes [31]. Sequencing of RNA (RNA-seq) is an emerging method of transcriptomic analysis which overcome the limitations of the hybridization-based approaches such as microarrays [32]. Unlike the microarray technology, which quantifies the intensity of the hybridization with a probe, RNA-seq measures the discrete number of transcripts that are aligned to a particular sequence. RNA-seq allows the unbiased detection of low-expressed genes, alternative splice variants, and novel transcripts, making this technology very suitable for biomarker discovery [33]. Metabolomics is an emerging field of -omics which is specialized in the analysis of small metabolites (<1500 daltons) that reflect the 'fingerprint' of the biochemical activity of the cell. Profiling of plasma metabolites may therefore shed light on the complex pathophysiological changes occurring during adipose tissue expansion. It has been described that different profiles of circulating lipids can predict the risk of cardiovascular disease [34] and insulin resistance [35]. Interestingly, a recent work reported that fatty acids released from necrotic adipocytes can act as powerful adjuvants for the immune response [36].

Additionally, ectopic deposition of lipids leads to an inflammatory response in the tissues where the deposition occurs [16,17]. This response is specially important in the visceral adipose where adipocytes anatomically associate with lymph nodes [37]. Inflammation drives the recruitment of other immune cells and activation of resident M1 macrophages which, in turn, produce various proinflammatory mediators (TNFa, IL-6, NOS) perpetuating the inflammation [38]. This phenotype can be reversed by IL-4-producing eosinophils able to keep macrophages in a non-inflamatory M2-phenotype [18]. It is hence of paramount importance incorporate in our project multiplexed flow cytometry to determine populations of immune cells within the adipose tissue and their pro- or anti-inflammatory phenotype. We posit that immune cell characterization coupled to gene expression information will enrich our dataset for developing multilevel biomarkers.

Integration of the data generated by high throughput technologies using mathematical models presents quite a few challenges. Unlike classical biomarkers (e.g. LDL for cardiovascular disease or HER2 for breast cancer), omics-based tests can not be explained simply on their biological basis. Omics-based biomarkers are generated by measuring many more variables and using multivariate statistical models. These models are very likely to be overadjusted so can result in a score that works well in the samples used to develop the test, but inaccurately in other samples [39]. In our study, the assessment of biomarker effectiveness for prediction will be separated from biomarker discovery. To obtain an unbiased estimation, the performance of the biomarker will be validated in another ongoing cohort of replication. Limitation for the validity of the biomarker might be the homogeneity in both test and validation cohorts, all white individuals from Spain. This phenomenon might weaken biomarker's predictive ability in other populations and would warrant to test the performance across population subgroups.

In summary, there is currently no clinically useful screening method to predict which obese individuals will develop metabolic derangements, specially diabetes and cardiovascular disease. Indeed, available and emerging therapies to prevent obesity cannot be targeted to those high-risk individuals who likely would benefit the most. This study will provide biomarkers based in high throughput techniques (RNA-seq, metabolomics, multiplexed cytometry) to detect impaired subcutaneous fat expandability, the underlying cause of pathologic obesity. To minimize the risk of false positive rates and increase their predictive performance we will use a validation cohort. This study will also provide mechanistic evidence that promoting fat expansion might be a suitable therapy to reduce metabolic complications associated with positive energy balance characteristic of Westernized societies.

## Ethical aspects

The study is conducted in accordance with the Declaration of Helsinki and good clinical practice guidelines. All participants must provide written informed consent. This study protocol was approved on 10 December 2014 (ref. #20/2014) by the Regional Institutional Review Board of Aragón (CEIC-A), Spain.

## References

[1] Haffner SM (2006). Relationship of Metabolic Risk Factors and Development of Cardiovascular Disease and Diabetes. Obesity.

[2] Whitlock G, Lewington S, Sherliker P, Clarke R, Emberson J, Halsey J (2009). Body-mass index and cause-specific mortality in 900 000 adults: collaborative analyses of 57 prospective studies. Lancet.

[3] Berrington de Gonzalez A, Hartge P, Cerhan JR, Flint AJ, Hannan L, MacInnis RJ (2010). Body-Mass Index and Mortality among 1.46 Million White Adults. N Engl J Med.

[4] Louie SM, Roberts LS, Nomura DK (1831). Mechanisms linking obesity and cancer. Biochim Biophys Acta.

[5] Karelis AD, Faraj M, Bastard J-P, St-Pierre DH, Brochu M, Prud’homme D (2005). The Metabolically Healthy but Obese Individual Presents a Favorable Inflammation Profile. J Clin Endocrinol Metab.

[6] Stefan N, Kantartzis K, Machann J, Schick F, Thamer C, Rittig K (2008). Identification and characterization of metabolically benign obesity in humans. Arch Intern Med.

[7] Wildman RP, Muntner P, Reynolds K, McGinn AP, Rajpathak S, Wylie-Rosett J (2008). The obese without cardiometabolic risk factor clustering and the normal weight with cardiometabolic risk factor clustering: prevalence and correlates of 2 phenotypes among the US population (NHANES 1999–2004). Arch Intern Med.

[8] Hamer M, Stamatakis E (2012). Metabolically Healthy Obesity and Risk of All-Cause and Cardiovascular Disease Mortality. J Clin Endocrinol Metab.

[9] Shea JL, Randell EW, Sun G (2011). The prevalence of metabolically healthy obese subjects defined by BMI and dual-energy X-ray absorptiometry. Obesity.

[10] Cristancho AG, Lazar MA (2011). Forming functional fat: a growing understanding of adipocyte differentiation. Nat Rev Mol Cell Biol.

[11] Zuk PA, Zhu M, Ashjian P, De Ugarte DA, Huang JI, Mizuno H (2002). Human adipose tissue is a source of multipotent stem cells. Mol Biol Cell.

[12] Sethi JK (2010). Activatin’ human adipose progenitors in obesity. Diabetes.

[13] Nishimura S, Manabe I, Nagasaki M, Hosoya Y, Yamashita H, Fujita H (2007). Adipogenesis in obesity requires close interplay between differentiating adipocytes, stromal cells, and blood vessels. Diabetes.

[14] Gray SL, Vidal-Puig AJ (2007). Adipose Tissue Expandability in the Maintenance of Metabolic Homeostasis. Nutr Rev.

[15] Laclaustra M, Corella D, Ordovas JM (2007). Metabolic syndrome pathophysiology: the role of adipose tissue. Nutr Metab Cardiovasc Dis.

[16] Xu H, Barnes GT, Yang Q, Tan G, Yang D, Chou CJ (2003). Chronic inflammation in fat plays a crucial role in the development of obesity-related insulin resistance. J Clin Invest.

[17] Cinti S, Mitchell G, Barbatelli G, Murano I, Ceresi E, Faloia E (2005). Adipocyte death defines macrophage localization and function in adipose tissue of obese mice and humans. J Lipid Res.

[18] Wu D, Molofsky AB, Liang H-E, Ricardo-Gonzalez RR, Jouihan HA, Bando JK (2011). Eosinophils sustain adipose alternatively activated macrophages associated with glucose homeostasis. Science.

[19] Lee Y-H, Chen S-Y, Wiesner RJ, Huang Y-F (2004). Simple flow cytometric method used to assess lipid accumulation in fat cells. J Lipid Res.

[20] Miranville A, Heeschen C, Sengenès C, Curat CA, Busse R, Bouloumié A (2004). Improvement of postnatal neovascularization by human adipose tissue-derived stem cells. Circulation.

[21] Boulet N, Estève D, Bouloumié A, Galitzky J (2013). Cellular heterogeneity in superficial and deep subcutaneous adipose tissues in overweight patients. J Physiol Biochem.

[22] Folch J, Lees M, Sloane-Stanley GH (1957). A simple method for the isolation and purification of total lipids from animal tissues. J Biol Chem.

[23] Krotkiewski M, Björntorp P, Sjöström L, Smith U (1983). Impact of obesity on metabolism in men and women. Importance of regional adipose tissue distribution. J Clin Invest.

[24] Janssen I, Katzmarzyk PT, Ross R (2004). Waist circumference and not body mass index explains obesity-related health risk. Am J Clin Nutr.

[25] Lin H-H, Lee J-K, Yang C-Y, Lien Y-C, Huang J-W, Wu C-K (2013). Accumulation of epicardial fat rather than visceral fat is an independent risk factor for left ventricular diastolic dysfunction in patients undergoing peritoneal dialysis. Cardiovasc Diabetol.

[26] Pinnick KE, Nicholson G, Manolopoulos KN, McQuaid SE, Valet P, Frayn KN (2014). Distinct developmental profile of lower-body adipose tissue defines resistance against obesity-associated metabolic complications. Diabetes.

[27] Seidell JC, Bakker CJ, van der Kooy K (1990). Imaging techniques for measuring adipose-tissue distribution--a comparison between computed tomography and 1.5-T magnetic resonance. Am J Clin Nutr.

[28] Shen W, Punyanitya M, Wang Z, Gallagher D, St-Onge M-P, Albu J (2004). Visceral adipose tissue: relations between single-slice areas and total volume. Am J Clin Nutr.

[29] Sottier D, Petit J-M, Guiu S, Hamza S, Benhamiche H, Hillon P (2013). Quantification of the visceral and subcutaneous fat by computed tomography: interobserver correlation of a single slice technique. Diagn Interv Imaging.

[30] Biomarkers Definitions Working Group (2001). Biomarkers and surrogate endpoints: preferred definitions and conceptual framework. Clin Pharmacol Ther.

[31] Montastier E, Villa-Vialaneix N, Caspar-Bauguil S, Hlavaty P, Tvrzicka E, Gonzalez I (2015). System model network for adipose tissue signatures related to weight changes in response to calorie restriction and subsequent weight maintenance. PLoS Comput Biol.

[32] Wang Z, Gerstein M, Snyder M (2009). RNA-Seq: a revolutionary tool for transcriptomics. Nat Rev Genet.

[33] Han H, Jiang X (2014). Disease Biomarker Query from RNA-Seq Data. Cancer Inform.

[34] Imamura F, Lemaitre RN, King IB, Song X, Lichtenstein AH, Matthan NR (2012). Novel circulating fatty acid patterns and risk of cardiovascular disease: the Cardiovascular Health Study. Am J Clin Nutr.

[35] Mozaffarian D, Cao H, King IB, Lemaitre RN, Song X, Siscovick DS (2010). Circulating palmitoleic acid and risk of metabolic abnormalities and new-onset diabetes. Am J Clin Nutr.

[36] Tynan GA, Hearnden CH, Oleszycka E, Lyons CL, Coutts G, O’Connell J (2014). Endogenous Oils Derived From Human Adipocytes Are Potent Adjuvants That Promote IL-1α-Dependent Inflammation. Diabetes.

[37] Mironov VA, Gusev SA, Baradi AF (1979). Mesothelial stomata overlying omental milky spots: Scanning electron microscopic study. Cell Tissue Res.

[38] Schipper HS, Prakken B, Kalkhoven E, Boes M (2012). Adipose tissue-resident immune cells: key players in immunometabolism. Trends Endocrinol Metab.

[39] Micheel CM, Nass SJ, Omenn GS (2012). Evolution of Translational Omics: Lessons Learned and the Path Forward.

